# Tools to support evidence-informed public health decision making

**DOI:** 10.1186/1471-2458-14-728

**Published:** 2014-07-18

**Authors:** Jennifer Yost, Maureen Dobbins, Robyn Traynor, Kara DeCorby, Stephanie Workentine, Lori Greco

**Affiliations:** 1 School of Nursing, Faculty of Health Sciences, McMaster University, 1200 Main St. W, Hamilton, Ontario, Canada; 2 Health Promotion, Chronic Disease & Injury Prevention, Public Health Ontario, 480 University Avenue, Suite 300, Toronto, Ontario, Canada

**Keywords:** Evidence-informed decision making, Knowledge translation and exchange, Knowledge broker, Public health, Tools

## Abstract

**Background:**

Public health professionals are increasingly expected to engage in evidence-informed decision making to inform practice and policy decisions. Evidence-informed decision making involves the use of research evidence along with expertise, existing public health resources, knowledge about community health issues, the local context and community, and the political climate. The National Collaborating Centre for Methods and Tools has identified a seven step process for evidence-informed decision making. Tools have been developed to support public health professionals as they work through each of these steps. This paper provides an overview of tools used in three Canadian public health departments involved in a study to develop capacity for evidence-informed decision making.

**Methods:**

As part of a knowledge translation and exchange intervention, a Knowledge Broker worked with public health professionals to identify and apply tools for use with each of the steps of evidence-informed decision making. The Knowledge Broker maintained a reflective journal and interviews were conducted with a purposive sample of decision makers and public health professionals. This paper presents qualitative analysis of the perceived usefulness and usability of the tools.

**Results:**

Tools were used in the health departments to assist in: question identification and clarification; searching for the best available research evidence; assessing the research evidence for quality through critical appraisal; deciphering the ‘actionable message(s)’ from the research evidence; tailoring messages to the local context to ensure their relevance and suitability; deciding whether and planning how to implement research evidence in the local context; and evaluating the effectiveness of implementation efforts. Decision makers provided descriptions of how the tools were used within the health departments and made suggestions for improvement. Overall, the tools were perceived as valuable for advancing and sustaining evidence-informed decision making.

**Conclusion:**

Tools are available to support the process of evidence-informed decision making among public health professionals. The usability and usefulness of these tools for advancing and sustaining evidence-informed decision making are discussed, including recommendations for the tools’ application in other public health settings beyond this study. Knowledge and awareness of these tools may assist other health professionals in their efforts to implement evidence-informed practice.

## Background

Systematically incorporating research evidence in program planning and policy decision making supports the provision of high-quality, effective, and efficient health services. This further ensures a more responsible use of the financial and human resource investments that are made in healthcare and in public health [1-3]. As such, public health professionals are increasingly expected to engage in evidence-informed decision making (EIDM). EIDM involves using research evidence with public health expertise, resources, and knowledge about community health issues, local context, and political climate to make policy and programming decisions [4].

Efforts are growing to promote EIDM within the public health sector in Canada [5-12]. To support such efforts, the National Collaborating Centre for Methods and Tools (NCCMT) has developed a seven step process to guide public health professionals through EIDM. This process includes: 1) defining the question, problem or issue; 2) searching for the best available research evidence; 3) assessing the quality of the evidence; 4) deciphering the ‘actionable message(s)’ from the evidence; 5) tailoring messages to the local context to ensure their relevance and suitability; 6) deciding whether and planning how to implement the evidence in the local context; and 7) evaluating the effectiveness of implementation efforts [13].

However, barriers to supporting, advancing, and sustaining EIDM exist at both individual and organizational levels [10,14,15]. The social, political, and historical context of public health practice and decision-making can also hinder the optimal use of evidence [10,16]. For example, the literature suggests that without an organized and methodical process for applying research evidence to decision making, the evidence can be selectively used to justify a decision that has already been made for other tactical or political reasons [16-18]. At an organizational level, barriers include a general resistance to change, limited access to evidence, unsupportive communication and organizational structures, heavy workloads, and frequent public health crises (e.g. outbreaks, environmental disasters) that require urgent attention [10,16]. Limited knowledge and skills to access, interpret, evaluate, and synthesize research evidence are additional barriers to EIDM at an individual level [3,17].

Conversely, EIDM can be facilitated by supportive infrastructure and organizational roles. Organization-level facilitators include strong leadership, a vision and commitment to EIDM, a receptive workforce culture, and committing time and financial resources to support EIDM [9-11,19,20]. The development of specific positions, such as Knowledge Brokers (KBs) or contracts with external KBs who are responsible for building capacity and supporting the use of evidence among public health professionals, helps establish an organizational climate that is supportive of research use [20-22]. EIDM is further advanced by improving access to research and library services, supporting the use of knowledge management tools that actively share relevant research evidence with users, and involving organizations in research activities that support collaboration between researchers and decision makers [1,9,10,14,15,17,20,23]. Individual-level facilitators include training and continuing education in EIDM and its associated knowledge and skill set [9,20].

Tools (guidelines, templates, checklists, assessment criteria, etc.) have been developed by various organizations for specific audiences, including public health, to support EIDM [13,24,25]. The use of such tools can help build health professionals’ skills and can assist them in appraising, synthesizing and applying research findings [1,9,24]. Previous studies have shown that a KB can play a key role in providing assistance in identifying, revising or creating applicable tools to further support engagement in EIDM at individual and organization levels [26].

The purpose of this paper is to report on the use of tools used by three Canadian public health departments in a study assessing the effectiveness of a KB-delivered knowledge translation and exchange (KTE) intervention*.* We describe the tools used to support steps in the EIDM process, evaluate their usability through qualitative analysis, and recommend their application beyond this study to the broader field of public health.

## Methods

### Study design

We partnered with three Ontario public health departments on a Canadian Institutes of Health Research (CIHR) ‘Partnerships for Health System Improvement’ (PHSI) grant (FRN 101867) to evaluate the effectiveness of KTE interventions to enhance capacity for and facilitate organizational contexts conducive to EIDM. This study received ethics approval from the McMaster University Research Ethics Board and the ethics boards of each participating health department. Using a case study design, we tailored a 22-month KTE intervention to the unique needs of each health department (Case A, Case B, Case C). The main strategy or component of each tailored intervention included a KB (authors LG and KD, with assistance from RT) working through the steps of EIDM [13] with selected public health professionals, including specialists (e.g. epidemiologists, consultants, Research and Policy Analysts (RPAs), dieticians, and nutritionists), management, and front line staff (e.g. Public Health Nurses, Health Promotion Officers, Public Health Inspectors, and dental professionals). Table 1 provides a description of the tailored intervention and outcomes for each Case. A more in depth discussion of the KTE intervention implemented at each of the three health departments has been submitted for publication and additional results are also expected to be published.

**Table 1:** Description of tailored interventions, data collection, and outcomes for each Health Department

	**Case A**	**Case B**	**Case C**
**Context**	Large, diverse population served; leadership had vision for EIDM; EIDM a strategic priority, with committed resources.	Large, urban centre served; leadership strongly committed to EIDM; manager ‘champion’ for EIDM; EIDM a strategic priority	Mid-size urban/rural mix served; leadership committed to EIDM, with additional support from executive.
**Intervention period**	September 2010 – June 2012	April 2011 – February 2013	April 2011 – December 2012
**Intervention strategies**	KB on site, 2 days/week:	KB on/off site, 2 days/week:	KB on/off-site, 2 days/week:
	- Provided workshop training for all staff;	- Provided introductory workshops to all consultants;	- Provided department-wide EIDM training;
	- Participated in intra-department presentations;	- Provided EIDM training for all staff in one directorate;	- Advised Research Knowledge & Exchange Committee on creation of EIDM guidebook;
	- Mentored staff teams through rapid evidence reviews;	- Mentored staff teams through rapid evidence reviews;	- Mentored staff teams through rapid evidence reviews;
	- Provided one-on-one consulting;	- Provided one-on-one consulting;	- Provided one-on-one consulting;
	- Regularly met with and presented to senior management.	- Advised senior management;	- Advised executive on EIDM Policy & Procedure.
		- Advocated for staff time to be allocated to EIDM.	

*Abbreviations*: *EIDM* evidence-informed decision making, *KB* Knowledge Broker.

### Data collection

Quantitative and qualitative data were collected to determine the impact of the intervention on knowledge, capacity, and behaviour for EIDM and the contextual factors that facilitated or impeded impact within each health department. Here we discuss the data collection strategies relevant to the qualitative analysis presented in this paper. This discussion adheres to the RATS guidelines for reporting qualitative studies [27]. The KBs delivering the intervention maintained a reflective journal to track meetings, observations, and reflections of their experiences in each of the health departments. Organizational documents were also collected. These included: strategic plans, internal communications related to EIDM (meeting minutes), policies and procedures related to the sharing and integration of EIDM, existing tools to facilitate the implementation of EIDM, and existing write-ups of literature reviews.

A purposive sample of senior management and public health professionals involved in the intervention were identified by the KB and a health department liaison to the research team. One member of the research team (RT) invited these staff, via email, to participate in a telephone interview. All staff who agreed to participate provided informed consent. One member of the research team (RT) conducted each telephone interview, lasting approximately 20–40 minutes, at baseline and follow-up using a semi-structured interview guide. At baseline, management and key contacts involved in supporting the research study were interviewed to understand the current organizational environment. At follow-up, staff that had been intensively involved in the intervention and additional management were interviewed. Participants were asked to reflect on the intervention and EIDM process at their respective health department and identify what they thought went well, including what resources or supports were helpful, and if they thought their colleagues were aware that these resources were available to them. They were also asked if any supports were “missing” or if they had suggestions for how the process could be improved. Data collection, via interviews, was considered complete when all identified staff had either declined to participate or were interviewed.

### Data analysis

All data collected throughout the intervention (baseline to follow-up) were analysed for this paper in order to understand the change in organizational use of tools. Interviews were recorded and transcribed verbatim, with light editing to remove fillers (“ums”, “ahs”), ambient sounds, non-verbal communication, and all identifying information. NVivo 9 was used for data management and coding. Two authors (RT, KD) independently coded several interview transcripts and journal entries based on an initial coding structure derived from the McKinsey 7-S Model [28-30], a framework used to help guide the study design. The authors compared their coding and further refined the structure as themes emerged using a constant comparative process [31]. One author (RT) applied the refined coding structure to analyze remaining data from all sources. Regular meetings were held with research team members involved in qualitative analysis (RT, KD, MD, and an additional co-investigator) to discuss any issues and proposed revisions to the coding scheme. The team discussed and came to consensus on any new themes. Organizational documents were reviewed and data relevant to the types of tools and how these tools were used within the health departments was extracted. The data was then reviewed by members of the research team and the KBs and presented back to key contacts at the health departments to confirm accuracy.

### Tools for EIDM

A variety of tools to support the steps of EIDM were used within the three health departments. New tools were created and existing tools were adapted to meet the health departments’ needs; several tools were formally adopted into health department policies and procedures. A number of tools were developed in Case A as part of an Executive Training for Research Application (EXTRA) Fellowship project of one senior manager [18,32]. Here we describe the tools that were created, adapted, used, and adopted at each of the health departments, organized by step of the EIDM process. Additional file 1 provides a succinct description of the tools and how they were used in the health departments, as well as identifies the developer of the tool and the format in which they are available. Table 2 provides an abridged version of Additional file 1.

**Table 2:** Tools to support evidence-informed decision making (EIDM)*

**Step**	**Tool**	**Description**	**Format**
**1. DEFINE**	**Developing an Efficient Search Strategy**	→ Assists users in turning a practice-based issue into an answerable, searchable question and identifying key terms to facilitate an efficient search [33].	Word document
	**Developing a Conceptual Model**	→ Guides users through the process of visually depicting the practice-based issue and question [18,37].	Word document
**2. SEARCH**	**6S Pyramid**	→ Guides users through searching published literature, moving from the most synthesized evidence to single studies [39].	Word document
	**Resources to Guide & Track Your Search**	→ Enables users to track the results of a search, and includes links to searchable databases [40,41].	Word document
	**Keeping Track of Search Results: A Flowchart**	→ Enables users to document the results of a search [42].	PowerPoint document
**3. APPRAISE**	**AGREE II Instrument**	→ Guides users through critical appraisal of practice guidelines [43-45].	PDF document
	**AMSTAR Tool**	→ Guides users through critical appraisal of syntheses [47-50].	Online, PDF document
	**Quality Assessment Tool**	→ Guides users through critical appraisal of syntheses [46].	PDF document
	**Critical Appraisal Skills Programme (CASP) Tools**	→ Guides users through critical appraisal of multiple study designs (syntheses, intervention studies, cohort studies, case–control studies, diagnostic studies, economic evaluations, clinical prediction rules, qualitative studies) [51].	PDF document
	**Scottish Intercollegiate Guidelines Network**	→ Guides users through critical appraisal of multiple study designs (syntheses, intervention studies, cohort studies, case–control studies, diagnostic studies, and economic evaluations) [52].	Word document
	**Quality Assessment Tool for Quantitative Studies**	→ Guides public health users through critical appraisal of multiple quantitative study designs (intervention studies, case–control studies, cohort studies, interrupted time series) [56].	PDF document
	**Critical Review Form Qualitative Studies**	→ Guides users through critical appraisal of qualitative studies [57,58].	PDF document
**4. SYNTHESIZE**	**Data Extraction for Systematic Reviews**	→ Provides a table-format template for users to extract relevant information from systematic reviews [18,59].	Word document
**5. ADAPT**	**Applicability & Transferability of Evidence Tool**	→ Guides users through the process and criteria for evaluating the applicability and transferability of the evidence to local contexts [65].	PDF document
	**Rapid Review Report Structure**	→ Guides users through writing the results of a Rapid Evidence Review [18,66].	PDF document
**6. IMPLEMENT**	**Knowledge Translation Planning Tool**	→ Guides users through planning, implementing, and evaluating plans for knowledge translation [68].	PDF document
**7. EVALUATE**	**Managers’ Checklist**	→ Provides users with an outline or checklist of the key elements of the EIDM process [18,72].	Word document

*For more detail, see Additional file 1.

#### ***Tools for defining the question/problem/issue***

The *Developing An Efficient Search Strategy* tool was developed by Health Evidence – an organization that facilitates EIDM among public health professionals in Canada – to turn practice-based issues into answerable, searchable questions [33]. This tool provides users with a framework for articulating different types of questions. It includes an explanation and public health-related example of how to identify important components related to an issue, including population, intervention, comparison, and outcomes for quantitative questions and population and setting for qualitative questions [34]. All three health departments used this tool. Cases A and C adapted and adopted it within their formal procedure for conducting “rapid evidence reviews”, defined as a more accelerated or streamlined version of traditional systematic reviews [35,36]. Case A also decided to develop a conceptual model of the practice-based issue before embarking on a rapid evidence review. Supported through an EXTRA Fellowship, a senior manager in this health department created the *Developing a Conceptual Model* tool [32,37]. This tool identifies five basic steps to guide users through the process of developing a model, with examples of public health-related issues, and has undergone modifications based on user feedback [18].

#### ***Tools for searching for the best available research evidence***

The *6S Pyramid* was developed by Haynes et al. [38] to help users efficiently and effectively find the best available research evidence relevant to their defined question. The tool guides searches through six levels of resources, beginning with the most synthesized evidence and ending with single studies [38,39]. A related tool, the *Resources to Guide & Track your Search,* was created by Health Evidence to enable easy access to public health relevant databases and track search results [40,41]. For each level of the *6S Pyramid*, the *Resources to Guide & Track your Search* tool provides the names and links to searchable databases for public health evidence. The tool indicates whether the databases are publicly available and whether the evidence retrieved from these databases has been quality appraised. Cases A and B used this tool and Case C adopted it in their formal procedure for conducting rapid evidence reviews. Health Evidence created a third tool, *Keeping Track of Search Results: A Flowchart,* as a template for documenting search results [42]. This tool enables users to clearly track the total number of articles identified from different sources and focus in on the final number of relevant articles from a search. The completed tool can be appended to the final version of a report of a rapid evidence review to increase the transparency of the process. Cases A and C adopted this tool into their formal procedures for conducting rapid evidence reviews, although some modifications have occurred to address user feedback.

#### ***Tools for assessing the research evidence for quality through critical appraisal***

The health departments used a variety of critical appraisal tools to assess the methodological quality of various types of research evidence. The *Appraisal of Guidelines for Research and Evaluation (AGREE) II Instrument,* an internationally accepted and tested tool was used by all health departments to assess the methodological rigor of practice guidelines [43-45]. The AGREE II Instrument contains 23 items within six quality domains. Its internal consistency ranges between 0.64 and 0.89 and its inter-rater reliability has been reported as satisfactory. The instrument’s items have been validated by stakeholder groups [43-45]. The AGREE II instrument concludes by assigning an overall quality rating and recommendation for using (or not using) the guideline [43]. The following two tools were used to assess the quality of systematic reviews: Health Evidence’s *Quality Assessment Tool *[46] and *A Measurement Tool to Assess Systematic Reviews (AMSTAR) *[47,48]. Health Evidence’s *Quality Assessment Tool* assigns an overall quality rating based on 10 items. The tool is accompanied by a dictionary that provides definitions of terms and instructions for assessing each criterion [46]. *AMSTAR* was initially developed for syntheses of randomized controlled trials (RCTs) but the 11-criteria tool has since been applied to syntheses that include non-RCTs [47,48]. The tool has demonstrated construct validity and satisfactory inter-observer agreement, with reliability of the total score documented as excellent [48]. The group is now developing a version to assess the quality of syntheses that include observational studies [49]. Available in Japanese, French and Spanish, the *AMSTAR* tool has received an endorsement from the Canadian Agency for Drugs and Technologies in Health and has been cited approximately 200 times over the past three years [50]. The Critical Appraisal Skills Programme (CASP) [51] and Scottish Intercollegiate Guidelines Network (SIGN) [52] have also developed tools for the critical appraisal of syntheses, as well as for several single study designs. Users appraise evidence using the CASP tools by asking: 1) “Is the study valid?”; 2) “What are the results?”; and 3) “Are the results applicable to my needs?” [51]. Since the core checklists (syntheses and randomized controlled trials) were developed and piloted, the suite of CASP tools has been expanded and evaluated for suitability [53] and usefulness [54]. The validity of the tool for qualitative studies has also been evaluated [55]. The SIGN tool provides an overall quality rating based on internal validity criteria [52]. In addition to these tools, all three health departments used the Effective Public Health Practice Project’s (EPHPP) *Quality Assessment Tool for Quantitative Studies* to appraise single studies. The EPHPP tool provides an overall quality rating based on six individual quality domains [56]. Finally, the health departments used the *Critical Review Form - Qualitative Studies (Version 2.0)* to assess the methodological quality of qualitative studies based on the rigor of eight components [57]. This tool, and its accompanying guidelines for users, has demonstrated an agreement of 75% to 86% between two researchers [58].

#### ***Tools for interpreting evidence and forming recommendations for practice***

Case A developed the *Data Extraction for Systematic Reviews* tool to guide rapid evidence reviews as part of an EXTRA fellowship. This tool has been modified following user feedback [18,59]. Users can use this table template to organize and synthesize research evidence, specifically by extracting actionable messages and recommendations from retrieved articles [59,60]. Case C adapted and formally adopted the tool into their organizational documents.

#### ***Tools for deciding whether to use the evidence in the local context***

NCCMT’s *Applicability and Transferability of Evidence Tool (A&T Tool)* identifies several areas (feasibility, generalizability) to consider when determining if the evidence is relevant for the local setting and circumstances [61-64]. The tool had reported content validity and can be applied either when starting or eliminating programs and interventions [63-65]. Cases A and C adapted the *A&T Tool* and included it in their organizational documents for conducting rapid evidence reviews. Case A created the *Rapid Review Report Structure* tool as part of the EXTRA fellowship, and has continually modified the tool based on user feedback. The tool’s purpose is to guide the writing up of the results of a rapid evidence review, outlining recommendations, and identifying and assigning responsibilities for next actions. The *Rapid Review Report Structure* tool builds on the Canadian Foundation for Healthcare Improvement’s standard report format [18,66,67]. The tool includes one page of key messages, a 2-page executive summary, and a full report of no more than 20 pages. Case C subsequently adapted and formally adopted the adapted tool.

#### ***Tools for deciding and planning how to implement the message in the local context***

The *Knowledge Translation Planning Guide* provides direction on how to plan, implement, and evaluate plans for knowledge translation (KT) [68,69]. Case C adopted this tool into their formal organizational documents to guide the EIDM process. The tool and its accompanying guidebook provide information on integrating KT into specific research projects, a summary of key factors for assessing a KT plan, examples of hypothetical KT plans, and a checklist for reviewing KT plans [70].

#### ***Tools for evaluating the effectiveness of implementation efforts***

The final step in the EIDM process involves evaluating the effectiveness of implementing the evidence-informed practice, program, or policy decision. As mentioned above, the *Knowledge Translation Planning Tool* incorporates evaluation of whether the intervention achieved the anticipated results (program evaluation) and whether the implementation strategies were delivered as intended (process outcomes) [71]. In the “KT Impact & Evaluation” component, the *Knowledge Translation Planning Tool* asks users to identify important aspects of evaluation such as the expected result of the intervention, the indicators of practice change, and a measure of usefulness. Case A developed a tool during the EXTRA fellowship to evaluate whether the original goals of the rapid evidence review were met [18]. The *Manager’s Checklist *[72], which continues to be modified, outlines key elements of the EIDM process with space to record comments on each element. The tool can be used to assess the impact of the rapid evidence review on decisions and serve as a quick reference for future reviews.

## Results

### Experiences in using tools for EIDM

A total of 37 interviews were conducted throughout the intervention. Participants who agreed to be interviewed represented project/team staff and specialists (n = 14), managers/support staff (n = 16), and senior management (n = 7), with varied backgrounds (undergraduate, graduate, and M.D. degrees), length of time in public health (from 3 to 30 years), and the number of rapid evidence reviews (from 0 to 3) in which they were personally involved. Over 170 journal entries from the KBs’ reflective journals were also analyzed. Themes related to easing the process of EIDM, accessibility, and a role in increasing users’ confidence emerged related to the tools used in this study. Speculation about future use of the tools, ideas for new tools, and suggestions to improve existing tools were also discussed. Over 160 organizational documents were collected from the key contacts in each health department to confirm and augment data collected through interviews and journal entries, including the use, adaptation, and adoption of specific tools.

#### ***Easing the process of EIDM***

Participants interviewed generally agreed that the tools facilitated engagement in the EIDM process by increasing efficiency, providing a concrete process to follow, providing guidance on searching for research evidence, and documenting their work. They thought the tools provided structure to the EIDM process and kept them “on track.” The tools’ accompanying instructions systematically outlined what needed to be considered, ultimately allowing for improved efficiency. In her journal entries, the KB reflected on how tools such as Health Evidence’s *Quality Assessment Tool* could be used for training purposes. Using this tool, the KB led participants through examples of good and poor quality systematic reviews to gain experience in critical appraisal.

The theme of easing the process of EIDM was evident in health departments where there had not been a concrete process in place prior to the study. The tools helped to define a process that public health professionals could follow, which appeared to further facilitate engagement in that process.

*“… I think the process itself, that was laid out for us, was good in terms of… just having outlines, the databases, and the searches that we should go to, kind of the pyramid approach, the systematic, where we kind of focus, in there. I think that was really good and helpful. So the tools themselves were good. … we’ve never had anything kind of laid out before so I think that, in itself, was great.” –* Specialist

“… I think there are a number of [consultants] for whom this is very exciting and they feel like, ‘Finally! I’m getting the tools that I need to do the work that I think is the work that I’m supposed to be doing!’” – Manager

In her journal, the KB emphasized the importance of using tools and templates to document participants’ work and keep track of their progress while maintaining transparency and repeatability in their efforts. She reflected on how this concept of documentation was new for most participants in all three health departments so participants appreciated having templates such as the *Data Extraction for Systematic Reviews* and *Rapid Review Report Structure* to work from and tailor to their own needs. Participants interviewed also identified specific tools, such as the *Resources to Guide & Track Your Search,* as being critical for supporting their work.

“*I love the - and I keep saying this - I love the tool, ‘The Resources to Guide & Track Your Search’. That’s my favourite! … I have a favourite. So when I oriented the other staff, it was like, ‘if you hear nothing else, remember this tool!’”* - Specialist

#### ***Accessibility***

Participants interviewed reported that the tools were easily accessible; following their involvement in an evidence review, they knew exactly where to go to access the tools for future work. Several tools have been compiled and made formally available to staff at two of the health departments. One health department posted them on their library website and the other included them in a draft organizational guidebook for conducting evidence reviews. This draft was introduced to all staff who attended workshops on EIDM with the health department during the study. Participants interviewed also reflected on the value of being able to access and easily download many of the tools online, free of charge. Furthermore, they noted the ease of using and navigating the tools as being important to effectively maximize the use of the tools.

*“I really liked the tools through Health Evidence … being able to go into the websites to search for literature there. It seemed fairly timely. It was quite easy, actually.”* – Specialist

#### ***Increasing confidence***

Participants interviewed also discussed having confidence in the tools. Using tools, in which they had confidence, in turn promoted their trust in the results and recommendations of the rapid evidence reviews.

*“…it's just fantastic that access and some structure and some templates and processes are available to do it in a systematic way. … to have the confidence that these tools and templates and processes have been developed and checked out, our confidence in what we’re finding when we go through them is high…”* – Manager

Having the tools to support the EIDM process also increased the self-confidence staff had in their roles related to EIDM within the health department. The use of the tools at different steps in the EIDM process or rapid evidence review helped the staff identify the expectations within their role and the role of others in the health department.

“*…the process, I have to say, was well out-lined. We had sort of a package that was given to us and I looked at it a lot … there was a Managers’ Checklist that I was … tasked at doing so there were those pieces that helped to keep you on track when this is new, helped to see where you’re going and just getting confident in your role as this manager/supervisor.” -* Specialist

#### ***Future use of the tools***

Participants identified the tools as being relevant and timely to both their current and anticipated work. They expected to apply the tools to their work and projects occurring beyond the study.

*“I think the tools, the tools that your team brought, were really helpful, too. Those tools are something that we can always take back and use and apply to other projects and other work we do. So the tools I found to be quite excellent.”* - Specialist

Through the interviews it also became evident that those involved in the study thought it was important to share the tools with staff from the health department who were not involved in the KTE intervention. Participants discussed that sharing the tools could promote their continued use by both themselves and their peers. The KB further reflected that the participants she worked with were now recommending tools and templates to their colleagues.

#### ***The tools can be improved***

Areas for improving the tools were also identified. Some tools were cited as having a great amount of detail, while others could be enhanced by providing further description and instructions for use. The KB reflected on conflicting responses to Health Evidence’s *Quality Assessment Tool*, where some participants found the latter three quality criteria to be difficult to interpret. She noted that through discussion and support from the KB, participants were able to eventually understand and be comfortable using the tool and its associated dictionary. On the other hand, the KB reflected that participants preferred this tool over other critical appraisal tools, because the dictionary was “immensely helpful” and the *Quality Assessment Tool* assigns a numerical score, providing a clearer conclusion on study quality. The *Resources to Guide and Track your Search* tool was another tool that was identified as being difficult to use:

*“[A team member] stated that she did not like the Resources to Guide and Track your Search tool. She found that many of the links (not publically available) did not work and the process for using it took too long.” -* KB reflective journal entry

Several formatting improvements were also identified. Participants suggested changing the layout of the *Data Extraction for Systematic Reviews* tool from a vertical to horizontal format (which was completed during the PHSI study) to make the tool more user-friendly for extracting and organizing data from rapid evidence reviews. Participants also suggested that consistently using a Word format for the tools would improve the ability to complete the tool within the document itself, versus as a PowerPoint slide or PDF format.

A final area of improvement related to the consistency in how the tools were used in the rapid evidence review process by the team members and library services. The KB reflected on this challenge of bringing all staff to the same understanding of the value of pre-processed data and where to begin a search. One participant suggested that the tools need to be **“**adopted in a practical way” so that when the library assists a team in searching for evidence, the team can be confident that the search aligns with the principles of the *6S Pyramid*.

#### ***Ideas for new tools***

Finally, participants identified topic areas for tool development. Some managers reflected on whether there could be a tool specifically for knowledge transfer and change management that goes beyond the current scope of the *A&T Tool*. Suggestions were made that perhaps this tool could include a template for how to disseminate the messages as a result of a rapid evidence review to decision makers and stakeholders in a timely and meaningful way. The *Managers’ Checklist* was also cited as a very useful tool, both for completing the process and writing the final report. In addition, one specialist suggested that it may be helpful to have a more specific Specialists’ Checklist.

## Discussion

KTE interventions are being designed and implemented to address expectations for the use of research evidence in public health decision making and to overcome the individual, organizational, and contextual barriers to supporting, advancing, and sustaining EIDM [73]. The availability of tools and the role of a KB to provide the tools, mentoring staff through their use, were identified as being critical for facilitating staff learning and supporting the steps of EIDM in this case study.

A search of the literature on tools for EIDM revealed limited published evaluations of these types of tools. Electronic information services providing access to tools for EIDM in public health have been formally evaluated [74] and some instruments, such as the AGREE II [43-45], AMSTAR [48,75], CASP series [53-55], Critical Review Form Qualitative Studies version 2.0 [57,58], and the A&T tool [65], have been either evaluated, validated, and/or shown to be reliable. But evaluation and usability data across the spectrum of tools discussed here remains inconsistent. The usability and usefulness of these tools, illustrated by the qualitative results from our study, attempts to address this gap.

### Usability and usefulness

Despite varied and inter-related definitions, the concepts of usability and usefulness were viewed favourably among the tools [76-78]. Public health professionals in our study found that the tools for EIDM were useful in providing a clear, concrete process to follow, thereby increasing their efficiency in learning new concepts related to EIDM. Participants were able to engage more successfully with their work because the tools guided them closely through the process of EIDM, systematically outlining the inputs and outputs required for each step and maintaining their focus on the task or step at hand. The usefulness of the tools, as defined by Seffah, was illustrated in their “practical utility” to enable “users to solve real problems in an acceptable way” [78].

Engagement in the EIDM process was further facilitated by the accessibility of the tools, meaning tools were easy to locate (online or in the health department’s guidebook or intranet), quick and free to download or acquire, and easy to navigate and understand. Similar findings have been reported with respect to the relationship between accessibility and usability of interactive software systems [78] and information products [79]. Simply stated, usability is rated low when a product is difficult to access [79]. Usability criteria cited as being necessary for the accessibility of software applications also applies in our assessment of tools, including: flexibility or the ability to tailor the product to the user; a minimal number of steps to access and use; the provision of a dictionary or user guide for access and use; self-descriptiveness, in that the purpose of the product is clearly conveyed; efficient navigability; and finally, simplicity of the product and its means of access [78]. Our analysis indicated that even with a dictionary, some of the tools may still be confusing for users who, in our study, required the assistance of a KB to guide them through using the tool. If these tools are meant to be stand-alone products, the developers need to ensure they are user-friendly and self-explanatory.

The final theme to emerge from our examination of the usability and usefulness of EIDM tools was the tools’ role in increasing confidence; having confidence in the tools promoted staff trust in the products that resulted from using the tools. The utility of the tools was further evident in increasing staff understanding of the expectations of their roles with respect to EIDM and improving their self-confidence within those roles. Others have reported confidence in tools and templates [41], with users deeming them to be reliable and/or credible for supporting aspects of work associated with EIDM [77,79].

### Overcoming barriers, leveraging facilitators

The demonstrated usability and usefulness of the tools appeared to reduce barriers previously identified by public health professionals to engage in EIDM. An often cited individual barrier to EIDM is time [1,9,16,24]. Time was also identified in our study as a significant barrier for participants, but the tools were recognized as one means to overcome this. The tools were quickly and easily accessible and outlined a clear process to follow, reducing ambiguity concerning the requirements for each step and eliminating the need for organizations to create internal processes from scratch. For example, Robeson et al. [41] illustrated how *the 6S Pyramid* could reduce the amount of time a public health practitioner spends searching for evidence by encouraging them to begin at the highest, or most synthesized, level of evidence where the amount of relevant evidence is less and therefore more manageable, and already synthesized (and often appraised for quality) [41]. The *Resources to Guide and Track your Search* tool further improves efficiency, providing users with a direct link to several electronic databases. While extra time may be required to initially learn their appropriate use, these tools ultimately improve staff efficiency, reducing the demand on staff time.

A second individual-level barrier is limited capacity among public health staff to appraise, synthesize, and apply research findings in practice [16,80]. Use of the tools in our study facilitated individuals’ ability to systematically engage in the EIDM process and effectively learn the skills required for each step. Upon reflection, study participants commented on how they were better equipped for future engagement in EIDM and in sharing the tools and their learnings to improve their colleagues’ capacity for EIDM.

Organizational-level barriers to EIDM, as cited in the literature and observed in our own work, include unclear organizational values and expectations for EIDM and inadequate resources or infrastructure to access evidence [16,20]. To address the latter barrier, the health departments in our study either incorporated the tools into organizational documents and library intranet sites, or encouraged staff to access them from freely available and easily accessible online sources. Integrating the tools into organizational processes and widely promoting them among staff in turn helped solidify the value of EIDM and clarify organizational expectations. The tools used in this study were therefore central to the development of infrastructure and organizational capacity to support and encourage EIDM.

### Organizational strategy and context

This study indicates that using the tools can assist in developing infrastructure within the organization to support and encourage EIDM. As suggested by Bowen and colleagues, EIDM “requires a change in how business is done, and the environment in which this business is conducted” [16]. Case study work shows that changes including the implementation of new tools should be part of a larger organizational strategy [9,11]. Being explicit about EIDM capacity building as a long-term process allows adequate time to create, pursue and reach realistic goals, both for individuals and organizations [11]. The usability and usefulness of the tools can further assist in supporting a consistent and replicable organizational process for EIDM [20]. An organizational approach requires active KT strategies that provide access to research and the technical infrastructure that supports that access [9,20]. It is important to be realistic about the infrastructure needed to support access [11].

Work with EIDM tools as part of this project identified strategies that may be important for sustaining the use of these tools as part of an organization-wide EIDM strategy beyond a time-limited KTE intervention. As indicated by our findings, public health professionals who used the tools intended to continue to use the tools in their future work, and share the tools with colleagues. With the use of new tools comes a need to acknowledge that learning how to use and apply them takes time [11,81]. Use of an Intranet or organizational website that incorporates the library system can also promote the sharing of tools for EIDM within the health department, increasing awareness and promoting the accessibility of tools and resources, leading to the development of organizational infrastructure to support and encourage the EIDM process [20]. While change is taking place, organizations must be aware that individuals are being asked to make a major change in the way they work [82]. A supportive culture and context for this change is needed [9,18,83,84]. Careful consideration of context is required in developing strategies for implementing knowledge translation strategies [85-87]. Generating the necessary positive context depends on leadership at the highest level of the organization [9,11]. Management support and accountability is also needed to support employees [11,81,84]. When managers help employees to acknowledge EIDM as a part of their role, managers themselves make fewer mistakes, organizations learn more, and there is more innovation [88]. While strong senior leadership and management play a key role, it is also necessary that all staff recognize that everyone has responsibility for sharing knowledge so that learning can take place [88].

### Limitations

While this paper provides insights into the usefulness and usability of tools to support EIDM, some limitations should be noted. Data collection and analysis occurred simultaneously at baseline to help inform the tailored interventions. However, the majority of analysis occurred after follow-up data collection, restricting the ability of the researchers to conclude whether data saturation had occurred. The interviews were conducted by one member of the research team (RT), who also participated in enacting the role of the KB at one of the health departments. This may have introduced the possibility that participants provided responses that they thought to be socially desirable and hence especially positive. Lastly, all of the interviewees received the intensive KB-delivered KTE intervention or were involved in the study with a role of management support or key contact. Therefore views about the usefulness and usability of tools may be different among public health professionals who do not interact with a KB or are involved in these roles.

### Recommendations

While a number of tools have been described in this paper, additional tools and options for accessing them are also available. For example: the NCCMT facilitates access to methods and tools for EIDM through The Registry for Methods and Tools [89]; the UK National Health Service public health evidence section includes implementation tools specific to public health [90]; and the Public Health Agency of Canada’s Canadian Best Practices Portal provides information and tools for EIDM [25] and program planning [91]. The developers of these and other EIDM tools, as well as those facilitating the use of the tools in practice (i.e. researchers, decision makers), should be encouraged to collect and share information about how the tools have been used and perceptions of their usability and usefulness. These evaluation efforts have the ability to inform the refinement of existing tools and the development of new tools to support EIDM. While the best way to facilitate the uptake of these tools by public health professionals remains to be determined, this study illustrated that a KB, as part of a tailored KTE intervention, was able to facilitate the integration of the tools within the health departments, including sharing tools between the health departments. Although there is no single way to generate readiness for this type of organizational change [92], future efforts can look to what is known about facilitating positive context for uptake [9,11,83,84,86]. Future efforts should continue to identify the effectiveness of KTE interventions as part of a broader strategy for promoting the integration of tools that support the EIDM process in practice.

## Conclusion

Public health professionals are increasingly expected to use research evidence in decision making. Along with KTE interventions, tools are being designed and implemented to help public health professionals meet these expectations and engage in the seven-step process for EIDM. Using a KTE intervention delivered by a KB, this study demonstrated that the KB facilitated the sharing and integration of tools to support the EIDM process among three Ontario health departments. Findings illustrated that the tools used by public health professionals working within varied roles in the health departments were viewed as usable and useful. Use of the tools facilitated individuals’ ability to engage in the EIDM process in a systematic way, which in turn increased staff confidence in formulating recommendations for practice, program, and policy decisions. It also encouraged their future engagement in the EIDM process. Efforts should continue to promote the awareness and use of the tools to assist public health professionals in their efforts to incorporate research evidence in practice, program, and policy decisions.

## Abbreviations

EIDM: Evidence-informed decision making; NCCMT: National Collaborating Centre for Methods and Tools; KB: Knowledge Broker; KTE: Knowledge translation and exchange; CIHR: Canadian Institutes of Health Research; PHSI: Partnerships for Health System Improvement; FRN: Funding Reference Number; RPA: Research and Policy Analyst; EXTRA: Executive Training for Research Application; AGREE: Appraisal of Guidelines for Research and Evaluation; AMSTAR: A Measurement Tool to Assess Systematic Reviews; RCT: Randomized controlled trial; CASP: Critical Appraisal Skills Programme; SIGN: Scottish Intercollegiate Guidelines Network; EPHPP: Effective Public Health Practice Project; A&T: Applicability & Transferability; KT: Knowledge translation.

## Competing interests

The authors declare that they have no competing interests.

## Authors’ contributions

MD conceived of the study and contributed to the draft and revisions of the paper. JY prepared the first draft and revisions of the paper. RT and KD contributed to the draft and revisions of the paper. SW assisted with literature searching, background writing, and reference checking. LG contributed to initial revisions of the paper. MD, JY, RT, KD, and LG contributed to study implementation, data collection, and data analysis. All authors read and approved the final manuscript.

## Pre-publication history

The pre-publication history for this paper can be accessed here:

http://www.biomedcentral.com/1471-2458/14/728/prepub

## Supplementary Material

Additional file 1 **Tools for Evidence Informed Decision Making (EIDM).** Additional file 1 provides a succinct description of the tools and how they were used in the health departments, as well as identifies the developer of the tool and the format in which they are available [93]. Click here for file
