# Supplementary material for: Tools to support evidence-informed public health decision making
Source: BMC Public Health. 2014 Jul 18;14:728. doi: 10.1186/1471-2458-14-728 (PMC4223550; doi:10.1186/1471-2458-14-728)
Supplement: Additional file 1 — Tools for Evidence Informed Decision Making (EIDM). Additional file 1 provides a succinct description of the tools and how they were used in the health departments, as well as identifies the developer of the tool and the format in which they are available [93]. [file 1471-2458-14-728-S1.pdf]

Additional File 1: Tools for Evidence Informed Decision Making (EIDM)

Step in EIDM Process	Tool Description	Health Department Activity
**1. Define**	***Developing an Efficient Search Strategy*** [33]<br>**Developed by:** healthevidence.org<br>**Format:** Word document<br>**Description:**<br>→ Turn a practice-based issue into an answerable, searchable question.<br>→ Framework for quantitative questions is PICO/PECO (*P:* population relevant to the question; *I:* intervention (therapy, test, etc.); *E:* risk factor, disease, condition or harm exposed to; *C*: comparison (standard or routine intervention, alternative treatment/exposure, or no treatment/exposure); *O:* outcome of interest).<br>→ Framework for qualitative questions is PS [*P:* population relevant to the question; *S:* situation, circumstances, or conditions of experiences one wants to understand or describe.<br>→ *For 2. Search (below)*: Identify key terms to facilitate an efficient search; assist in documenting search strategies/terms.	**A** Adapted; used; formally adopted<br>**B** Used<br>**C** Adapted; used; formally adopted
	Developing a Conceptual Model [18,37]<br>**Developed by:** *Case A*<br>**Format:** Word document<br>**Description:**<br>→ Guides the process of visually depicting the issue and question.<br>→ Steps include: 1) define the practice issue; 2) identify your team; 3) review current knowledge of the issue, including if conceptual models have already been developed; 4) draw the conceptual model; and 5) verify conceptual model with stakeholders.	**A** Created; used; formally adopted<br>**B** -<br>**C** Adapted; used; formally adopted

**2. Search**	***6S Pyramid*** [39] *(See "Levels & Sources of Public Health Evidence")*<br>**Developed by:** DiCenso A, Bayley L, Haynes RB<br>**Format:** Word document<br>**Description:**<br>→ Six levels of resources that are regularly updated and have undergone a filtering process to include studies that are of higher quality.<br>→ Guides a search, starting with the most synthesized evidence, down the pyramid to single studies.<br>→ 6S: systems, summaries, synopses of syntheses, syntheses, synopses of studies, and studies.	**A** Used; formally adopted<br>**B** Used<br>**C** Used; formally adopted
	Resources to Guide & Track Your Search [40-41]<br>**Developed by:** healthevidence.org<br>**Format:** Word document<br>**Description:**<br>→ Tracks the results of a search that follows the framework of the 6S Pyramid.<br>→ Links to searchable databases for evidence related to public health.<br>→ Indicates whether databases are publicly available and whether the evidence retrieved from the database require quality assessment.	**A** Used<br>**B** Used<br>**C** Used; formally adopted
	Keeping Track of Search Results: A Flowchart [42]<br>**Developed by:** healthevidence.org<br>**Format:** PowerPoint document<br>**Description:**<br>→ Documents the results of searches.<br>→ Provides a quick "snapshot" of search results that is easy to share.	**A** Adapted; used; formally adopted<br>**B** Used<br>**C** Used; formally adopted

**3. Appraise**	***AGREE II Instrument*** [43]<br>**Developed by:** AGREE Next Steps Consortium<br>**Format:** PDF document<br>**Description:**<br>→ Critical appraisal of practice guidelines.<br>→ Six quality assessment domains and two overall assessment items (overall quality of and a recommendation to use the practice guideline).<br>→ Tool is reliable and valid; internal consistency ranges between 0.64 and 0.89, inter-rater reliability has been reported as satisfactory, and items have been validated by stakeholder groups [43-45].	**A** Used<br>**B** Used<br>**C** Used; formally adopted
	AMSTAR Tool [48]<br>**Developed by:** Shea BJ et al.<br>**Format:** Online and PDF document<br>**Description:**<br>→ Critical appraisal of syntheses; eleven quality assessment criteria.<br>→ Tool is reliable and valid; demonstrated construct validity and satisfactory inter-observer agreement, with reliability of the total score documented as excellent [48, 75].	**A** Used; formally adopted<br>**B** -<br>**C** -
	Quality Assessment Tool [46]<br>**Developed by:** healthevidence.org<br>**Format:** PDF document<br>**Description:**<br>→ Critical appraisal of syntheses.<br>→ Quality criteria include: clearly focused research question; provision of inclusion criteria; comprehensive search strategy; search strategy that covers an adequate number of years; rigour of studies included in the review is described; quality assessment of primary studies; transparency of quality assessments; appropriateness of combining study results is assessed; weighting; and interpretation of results.<br>→ Results in an overall quality rating out of 10 (8-10 is rated strong, 5-7 is rated moderate, and 1-4 is rated weak).	**A** Used; formally adopted<br>**B** Used<br>**C** Used; formally adopted

Critical Appraisal Skills Programme (CASP) Tools [51]

Developed by: Critical Appraisal Skills Programme

Format: PDF document

Description:

→ Critical appraisal of multiple study designs (syntheses, intervention studies, cohort studies, case-control studies, diagnostic studies, economic evaluations, clinical prediction rules, qualitative studies)

→ Proceeds in three steps. Step 1: Is the study valid? The user must decide whether the study was unbiased by evaluating its methodological quality using different criteria for validity for different types of questions (i.e. questions about intervention, diagnosis, etc.). Step 2: What are the results? Consider the certainty and clinical importance of the results. Step 3: Are the results applicable to my needs? If the evidence is valid and clinically important, as determined in Steps 1 and 2, now the user must decide if the evidence applies to the clinical question.

→ The core CASP checklists (randomised controlled trial and systematic review) were based on the Journal of the American Medical Association's original "Users' guides to the medical literature" [93] and piloted with health care practitioners Tools subsequently developed were developed and piloted by experts [54].

→ The tools were evaluated for suitability within a broad audience [53] and validity of tool for qualitative studies has been evaluated [31]. A survey of checklist users reiterated that the basic format continues to be useful and appropriate [93].

A Used; formally adopted

B Used

C Used; formally adopted

Scottish Intercollegiate Guidelines Network: Critical Appraisal Checklists [52]<br>**Developed by:** Scottish Intercollegiate Guidelines Network<br>**Format:** Word document<br>**Description:**<br>→ Critical appraisal of multiple study designs (syntheses, intervention studies, cohort studies, case-control studies, diagnostic studies, and economic evaluations).<br>→ Rate the quality on two domains: internal validity and overall assessment.<br>→ Items rated "yes", "no", "can't say" with criteria for rating provided.<br>→ Checklists are accompanied by a "Notes" worksheet.<br>→ Overall quality rating of low quality (either most criteria not met, or significant flaws relating to key aspects of study design; conclusions likely to change in the light of further studies), acceptable (most criteria met; some flaws in the study with an associated risk of bias; conclusions may change in the light of further studies), or high quality (majority of criteria met; little or no risk of bias; results unlikely to be changed by further research).	**A** Formally adopted<br>**B** -<br>**C** -
Quality Assessment Tool for Quantitative Studies [56]<br>**Developed by:** Effective Public Health Practice Project<br>**Format:** PDF document<br>**Description:**<br>→ Initially developed for public health.<br>→ Critical appraisal of different types of quantitative study designs (intervention studies, case-control studies, cohort studies, interrupted time series).<br>→ Rate the studies on six quality domains: selection bias, design, confounders, blinding, data collection methods, and withdrawals and dropouts.<br>→ Overall quality rating for the study of weak (two or more sections rated as weak), moderate (one section rated as weak), or strong (no sections rated as weak).	**A** Used<br>**B** -<br>**C** -

	Critical Review Form Qualitative Studies (Version 2.0) [57-58]<br>**Developed by:** Letts, L et al.<br>**Format:** PDF document<br>**Description:**<br>→ Critical appraisal of qualitative studies.<br>→ Domains: study purpose; relevant background literature; study design; sampling; data collection (descriptive clarity, procedural rigour); data analyses (analytical rigour, auditability, theoretical connections); overall rigour; conclusions/implications.<br>→ Tool is reliable; demonstrated an agreement of 75% to 86% between two researchers [58].	**A** Used; formally adopted<br>**B** -<br>**C** -
**4. Synthesize**	***Data Extraction for Systematic Reviews*** [18, 59]<br>**Developed by:** *Case A*<br>**Format:** Word document<br>**Description:**<br>→ The tool provides a table-format template for users to extract relevant information from systematic reviews included in rapid evidence reviews<br>→ The table includes information such as the reference, the quality rating, the methodological details (number and types of studies included, search strategy, inclusion/exclusion criteria), details about the interventions (setting, providers, target group, theoretical framework), the primary and secondary outcomes, the main results, and comments/limitations.	**A** Created; used<br>**B** -<br>**C** Adapted; used; formally adopted

**5. Adapt**	***Applicability & Transferability of Evidence Tool (A&T Tool)*** [65]<br>**Developed by:** National Collaborating Centre for Methods and Tools<br>**Format:** PDF document<br>**Description:**<br>→ Process and criteria for evaluating the applicability and transferability of the evidence to local context.<br>→ Items used to assess applicability (feasibility) include: political acceptability or leverage; social acceptability; available resources; and organizational expertise and capacity. Transferability (generalizability) is assessed by determining the characteristics of the target population, the magnitude of the health issue in the local setting, and the 'reach' of the intervention.<br>→ Tool demonstrates acceptable content validity [65].	**A**<br>**B**<br>**C**	Adapted; used; formally adopted<br>-<br>Adapted; used; formally adopted
	Rapid Review Report Structure [18,66]<br>**Developed by:** *Case A*<br>**Format:** PDF document<br>**Description:**<br>→ Guides writing the results of a rapid evidence review, outlining recommendations, and identifying and assigning responsibilities for next actions.<br>→ Builds on the Canadian Foundation for Healthcare Improvement's standard report format [67]: four to six key messages (1 page), an executive summary (2 pages), and a full report of findings (20 pages).	**A**<br>**B**<br>**C**	Created; used<br>-<br>Adapted; used; formally adopted

**6. Implement**<br>**7. Evaluate**	***Knowledge Translation Planning Tool*** [68]<br>**Developed by:** Barwick, M<br>**Format:** PDF document<br>**Description:**<br>→ Provides direction on how to plan, implement, and evaluate plans for knowledge translation.<br>→ Consists of 4 content areas: project partners; degree of partners' engagement; partners' roles; KT expertise on team; knowledge users; main messages; KT goals; KT method(s); KT process; KT impact and evaluation; resources; budget items related to the KT plan; and a description of the processes/procedures involved in implementing the KT strategies, considering retaining quality, fidelity, sustainability for practice or behaviour change strategies.<br>→ Accompanied by a Guidebook which is separated into four sections: background on the integration of KT into specific research project; a summary of key factors for consideration of assessing a KT plan; examples of hypothetical KT plans; and a checklist of key questions for use in reviewing KT plans.	**A** -<br>**B** -<br>**C** Used; formally adopted
	Manager's Checklist [18,72]<br>**Developed by:** *Case A*<br>**Format:** Word document<br>**Description:**<br>→ Outlines key elements of the EIDM process; users can record comments on each element.<br>→ Used to assess the impact of rapid evidence reviews on decisions; serve as a quick reference for future reviews.	**A** Created; used<br>**B** -<br>**C** -

Reference List

18. Ward M: **Evidence-informed decision making in a public health setting.** *Healthc Manage Forum* 2011, **24:** S8-16.

31. Hannes K, Lockwood C, Pearson A: **A comparative analysis of three online appraisal instruments' ability to assess validity in qualitative research.** *Qual Health Res* 2010, **20:** 1736-1743.

33. Health Evidence: **Developing an efficient search strategy** [http://www.healthevidence.org/practice-tools.aspx#PT2]

37. Region of Peel Public Health: **Step 1 - Developing a conceptual model - instructions and worksheet** [http://www.peelregion.ca/health/library/developing-model.asp]

39. DiCenso A, Bayley L, Haynes RB: **Accessing pre-appraised evidence: Fine-tuning the 5S model into a 6S model.** *Evid Based Nurs* 2009, **12:** 99-101.

40. Robeson P, Yost J: **Resources to Guide & Track your Search** [http://www.healthevidence.org/documents/practice-tools/HETools_ResourcesGuide&Track YourSearch_18.Mar.2013.doc]

41. Robeson P, Dobbins M, DeCorby K, Tirilis D: **Facilitating access to pre-processed research evidence in public health.** *BMC Public Health* 2010, **10:** 95.

42. Health Evidence: **Keeping track of search results: A flowchart** [http://www.healthevidence.org/practice-tools/HETools_KeepingTrackSearchResultsFlowchart_18.Mar.2013.ppt]

43. Brouwers MC, Kho ME, Bowman GP, Cluzeau F, Feder G, Fervers B, Graham ID, Grimshaw J, Hanna SE, Littlejohns P, Makarski J, Zitzelsberger L, AGREE Next Steps Consortium: **AGREE II: Advancing guideline development, reporting and evaluation in healthcare.** *Can Med Assoc J* 2010, **182:** E839-E842.

44. Brouwers MC, Kho ME, Browman GP, Burgers JS, Cluzeau F, Feder G, Fervers B, Graham ID, Hanna SE, Makarski J, AGREE Next Steps Consortium: **Development of the AGREE II, part 1: performance, usefulness and areas for improvement.** *CMAJ* 2010, **182:** 1045-1052.

45. Brouwers MC, Kho ME, Browman GP, Burgers JS, Cluzeau F, Feder G, Fervers B, Graham ID, Hanna SE, Makarski J, AGREE Next Step Consortium: **Development of the AGREE II, part 2: assessment of validity of items and tools to support application.** *CMAJ* 2010, **182:** E472-E478.

46. Health Evidence: **Quality assessment tool: Review articles** [http://www.healthevidence.org/documents/our-appraisal-tools/QA_tool&dictionary_18.Mar.2013.pdf]

48. Shea BJ, Hamel C, Wells GA, Bouter LM, Kristjansson E, Grimshaw J, Henry DA, Boers M: **AMSTAR is a reliable and valid measurement tool to assess the methodological quality of systematic reviews.** *J Clin Epidemiol* 2009, **62:** 1013-1020.

51. Critical Appraisal Skills Programme. **Appraising the evidence** [http://www.casp-uk.net/find-appraise-act/appraising-the-evidence/]

52. Scottish Intercollegiate Guidelines Network: **Critical appraisal: Notes and checklists** [http://www.sign.ac.uk/methodology/checklists.html]

53. National Collaborating Centre for Methods and Tools: **Critical appraisal tools to make sense of evidence** [http://www.nccmt.ca/registry/view/eng/87.html]

54. Critical Appraisal Skills Programme: **History** [http://www.casp-uk.net/#!history/cnxo]

56. Effective Public Health Practice Project: **Quality assessment tools for quantitative studies** [http://www.ephpp.ca/tools.html]

57. Letts L, Wilkins S, Law M, Stewart D, Bosch J, Westmorland M: **Critical Review Form – Qualitative Studies (Version 2.0)** [http://www.srs-mcmaster.ca/Portals/20/pdf/ebp/qualreview_version2.0.pdf]

58. **Occupational Therapy Evidence-Based Practice Research Group** [http://www.srs-mcmaster.ca/Default.aspx?tabid=630]

59. Region of Peel Public Health: **Step 4 - Data extraction for systematic reviews** [http://www.peelregion.ca/health/library/data-extraction.asp]

65. National Collaborating Centre for Methods and Tools: **Applicability and transferability of evidence tool (A&T tool)** [http://www.nccmt.ca/registry/view/eng/24.html]

66. Region of Peel Public Health: **Step 6 - Rapid review report structure** [http://www.peelregion.ca/health/library/report-structure.asp]

67. Canadian Health Services Research Foundation: **Communication notes: Reader-friendly writing - 1:3:25** [http://www.chsrf.ca/knowledge_transfer/communication_notes/comm_reader_friendly_writing_e.php]

68. Barwick M: **Scientist Knowledge Translation Plan Template - R™** [http://www.melaniebarwick.com/training.php]

72. Region of Peel Public Health: **Step 7 - Manager checklist** [http://www.peelregion.ca/health/library/manager-checklist.asp]

75. Shea BJ, Bouter LM, Peterson J, Boers M, Anderson N, Ortiz Z, Ramsay T, Bai A, Shukla VK, Grimshaw JM: **External validation of a measurement tool to assess systematic reviews (AMSTAR).** *PLoS Medicine* 2007, **2:** e1350.

93. Guyatt GH, Rennie D: **Users' guides to the medical literature.** *JAMA* 1993, **270:** 2096-2097.
